# Supplementary figures and images for: Unfolding and identification of membrane proteins in situ
Source: eLife. 2022 Sep 12;11:e77427. doi: 10.7554/eLife.77427 (PMC9531951; doi:10.7554/eLife.77427)

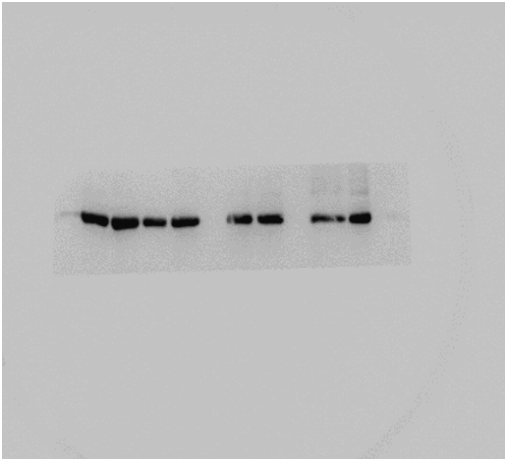

Supplement: Figure 6—figure supplement 1—source data 1. [file elife-77427-fig6-figsupp1-data1.zip › gels_images/Abottom.tif]

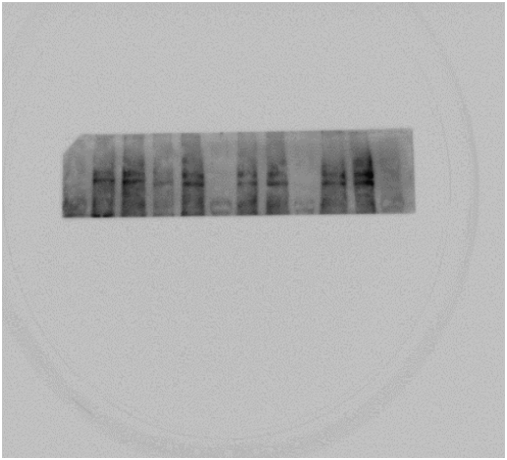

Supplement: Figure 6—figure supplement 1—source data 1. [file elife-77427-fig6-figsupp1-data1.zip › gels_images/Atop.tif]

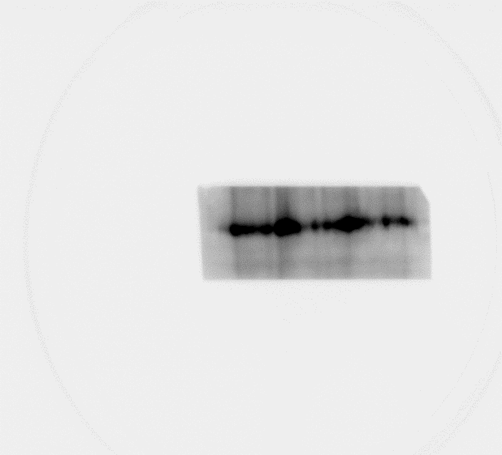

Supplement: Figure 6—figure supplement 1—source data 1. [file elife-77427-fig6-figsupp1-data1.zip › gels_images/Bbottom.tif]

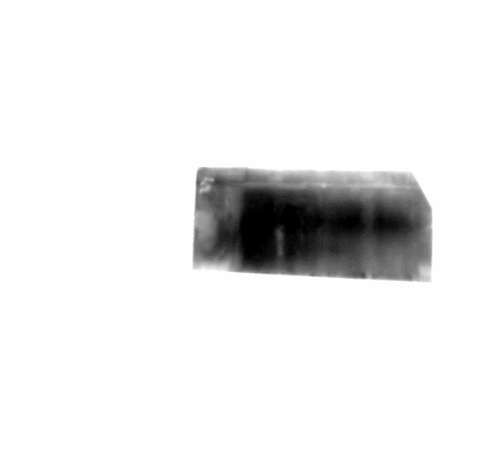

Supplement: Figure 6—figure supplement 1—source data 1. [file elife-77427-fig6-figsupp1-data1.zip › gels_images/Btop.tif]

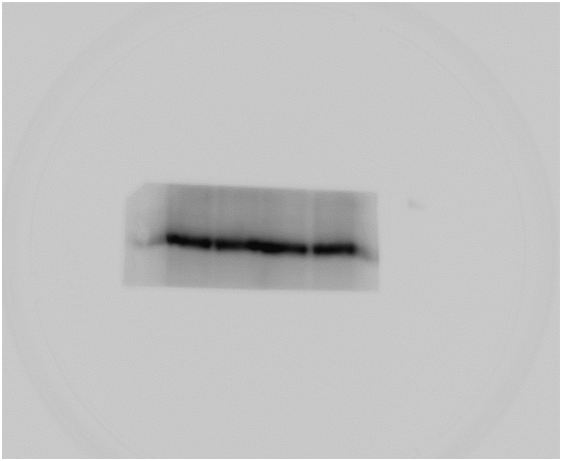

Supplement: Figure 6—figure supplement 1—source data 1. [file elife-77427-fig6-figsupp1-data1.zip › gels_images/Cbottom.tif]

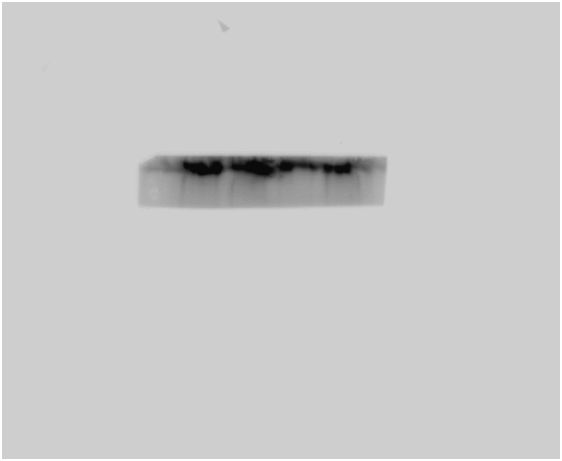

Supplement: Figure 6—figure supplement 1—source data 1. [file elife-77427-fig6-figsupp1-data1.zip › gels_images/Ctop.tif]

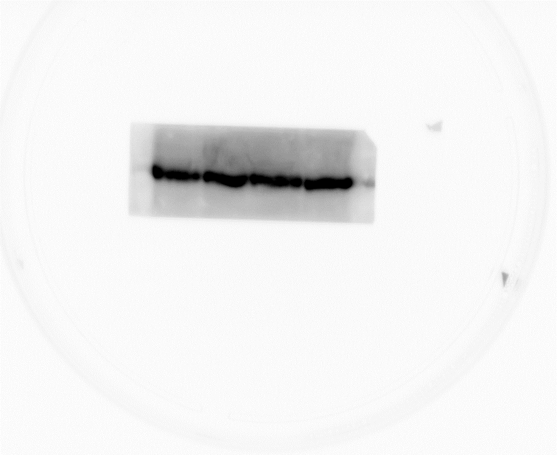

Supplement: Figure 6—figure supplement 1—source data 1. [file elife-77427-fig6-figsupp1-data1.zip › gels_images/Dbottom.tif]

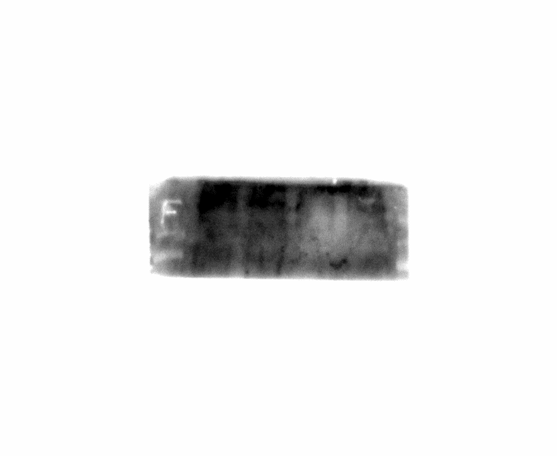

Supplement: Figure 6—figure supplement 1—source data 1. [file elife-77427-fig6-figsupp1-data1.zip › gels_images/Dtop.tif]
