# Supplementary material for: Unfolding and identification of membrane proteins in situ
Source: eLife. 2022 Sep 12;11:e77427. doi: 10.7554/eLife.77427 (PMC9531951; doi:10.7554/eLife.77427)
Supplement: Supplementary file 1. [file elife-77427-supp1.docx]

| \| lasmids \| Vector \| Provider \| Company \| \| --- \| --- \| --- \| --- \| \| peGFP-N1 \|  \| Prof. Guidalberto Manfioletti \|  \| \| mTMEM16A-GFP \| peGFP-N1 \| Prof. Anna Menini \|  \| \| mTMEM16F-GFP \| peGFP-N1 \| Prof. Anna Menini \|  \| \| 6xHis-N2B-mTMEM16F-GFP \| peGFP-N1 \|  \| GENEWIZ \| \| 6xHis-N2B-mTRPC1-GFP \| peGFP-N1 \|  \| GENEWIZ \| \| 6xHis-N2B-mTRPC5-GFP \| peGFP-N1 \|  \| GENEWIZ \| \| 6xHis-N2B-mTRPC6-GFP \| peGFP-N1 \|  \| GENEWIZ \| \| mCherry-Lifeact-7 \|  \| Prof. Michael Davidson \| Addgene \| |
| --- | --- | --- | --- | --- | --- | --- | --- | --- | --- | --- | --- | --- | --- | --- | --- | --- | --- | --- | --- | --- | --- | --- | --- | --- | --- | --- | --- | --- | --- | --- | --- | --- | --- | --- | --- | --- |
|  |
| **Table S1** |
| List of plasmids.  The cDNA of TRPC1/5/6 were from Miao Ling Plasmid Company. All the 6xHis-N2B-Protein-GFP construct is made by Genewiz company. |

| \| No \| Name \| Sequence(20bp) \| \| \| --- \| --- \| --- \| --- \| \|  \|  \|  \|  \| \| 1 \| plentiCRISPR V2-TRPC1- sgRNA1(mouse) \| ccgtaagcccacctgtaaga \| \| \|  \|  \| \|  \| \| 2 \| plentiCRISPR V2-TRPC1- sgRNA2(mouse) \| acgcttgtagcagaagggct \| \| \|  \|  \| \|  \| \| 3 \| plentiCRISPR V2-TRPC5- sgRNA1(mouse) \| attactctacgccatccgca \| \| \|  \|  \| \|  \| \| 4 \| plentiCRISPR V2-TRPC5- sgRNA2(mouse) \| ggagtgtgtatccagttcgg \| \| \|  \|  \| \|  \| \| 5 \| plentiCRISPR V2-TRPC6- sgRNA1(mouse) \| gcggcagacgattcttcgtg \| \| \|  \|  \| \|  \| \| 6 \| plentiCRISPR V2-TRPC6- sgRNA2(mouse) \| taaaggttatgtacggattg \| \| \|  \|  \| \|  \| \| 7 \| plentiCRISPR V2-TMEM16F- sgRNA1(mouse) \| agcgagcgttacctcctgta \| \| \|  \|  \| \|  \| \| 8 \| plentiCRISPR V2-TMEM16F- sgRNA2(mouse) \| ctctcgggtcaaataccaag \| \| \|  \|  \| \|  \| \| 9 \| plentiCRISPR V2-control-sgRNA \| tcttgagtttgtaacagctg \| \| |
| --- | --- | --- | --- | --- | --- | --- | --- | --- | --- | --- | --- | --- | --- | --- | --- | --- | --- | --- | --- | --- | --- | --- | --- | --- | --- | --- | --- | --- | --- | --- | --- | --- | --- | --- | --- | --- | --- | --- | --- | --- | --- | --- | --- | --- | --- | --- | --- | --- | --- | --- | --- | --- | --- | --- | --- | --- | --- | --- | --- | --- | --- | --- | --- | --- | --- | --- | --- | --- | --- | --- | --- | --- | --- | --- | --- | --- |
| **Table S2** |
| sgRNA Sequences.  The sgRNA sequences cloned into LentiCRISPR-V2 puro vector were purchased from Miao Ling Plasmid Company. |
