# Supplementary material for: Unfolding and identification of membrane proteins in situ
Source: eLife. 2022 Sep 12;11:e77427. doi: 10.7554/eLife.77427 (PMC9531951; doi:10.7554/eLife.77427)
Supplement: Supplementary file 2. [file elife-77427-supp2.docx]

| **Cell type** | **DRG** | **Hippocampus** | **Rod** | **Disc** | **NG108-15** | **bR** | **Sthk** | **ChR1** |
| --- | --- | --- | --- | --- | --- | --- | --- | --- |
| Number of animals* | 5 | 6 | 4 | 3 | na | na | na | na |
| Number of samples (experiemnts) | 8 | 10 | 15 | 6 | 14 | 5 | 5 | 1 |
| Number of cantilevers used | 8 | 10 | 15 | 6 | 14 | 5 | 5 | 1 |
| Total number of traces | 413468 | 301654 | 386128 | 106528 | 394118 | 35599 | 115155 | 18868 |
| Total number of non-flat traces (> 30pN at > 50nm) | 17211 | 32898 | 14910 | / | 34219 | / | / | / |
| Number of clusters | 22 | 38 | 18 | 26 | 17 | 3 | 7 | 7 |
| Number of traces clustered (survived the clustering quality filter) | 2365 | 1603 | 14910 | 1117 | 4403 | 196 | 1018 | 843 |
| Number of final clusters (after merging for similarity and cutoff)** | 15 | 10 | 8 | 5 | 11 | 1 | 2 | 3 |
| Total number of traces in final clusters *** | 1255 | 563 | 1039 | 703 | 1591 | 83 | 307 | 213 |
|  |  |  |  |  |  |  |  |  |
|  |  |  |  |  |  |  |  |  |
|  |  |  |  |  |  |  |  |  |
| *some primary cultures were used in consecutive experiments |  |  |  |  |  |  |  |  |
| **Merged clusters list present in Figure 1-figure supplement 9 |  |  |  |  |  |  |  |  |
| ***Number of traces in each final cluster is present in Figure 3 |  |  |  |  |  |  |  |  |

| **Table** |
| --- |
| Samples statistics |
